# Simple Vanilla Derivatives for Long-Lived Room-Temperature Polymer Phosphorescence as Invisible Security Inks

**DOI:** 10.34133/2021/8096263

**Published:** 2021-02-16

**Authors:** Yongfeng Zhang, Zhonghao Wang, Yan Su, Yan Zheng, Wenji Tang, Chaolong Yang, Hailong Tang, Lunjun Qu, Youbing Li, Yanli Zhao

**Affiliations:** ^1^School of Materials Science and Engineering, Chongqing University of Technology, Chongqing 400054, China; ^2^Division of Chemistry and Biological Chemistry, School of Physical and Mathematical Sciences, Nanyang Technological University, 21 Nanyang Link, Singapore 637371

## Abstract

Developing novel long-lived room-temperature polymer phosphorescence (RTPP) materials could significantly expand their application scope. Herein, a series of RTPP materials based on eight simple vanilla derivatives for security ink application are reported. Attributed to strong mutual hydrogen bonding with polyvinyl alcohol (PVA) matrix, vanilla-doped PVA films exhibit ultralong phosphorescence emission under ambient conditions observed by naked eyes, where methyl vanillate shows the longest emission time up to 7 s. Impressively, when vanilla-doped PVA materials are utilized as invisible security inks, and the inks not only present excellent luminescent emission stability under ambient conditions but also maintain perfect reversibility between room temperature and 65°C for multiple cycles. Owing to the unique RTPP performance, an advanced anticounterfeiting data encoding/reading strategy based on handwriting technology and complex pattern steganography is developed.

## 1. Introduction

Pure organic luminogens with room temperature phosphorescence (RTP) have attracted increasing attention owing to their unique material characteristics and the wide application potential in optoelectronic and biological fields including sensitive sensing [1–3], anticounterfeiting [4, 5], organic optoelectronics [6–8], and biological imaging [9–11]. To obtain efficient phosphorescent emission [12], many strategies based on the molecular structure design and spatial stacking have been proposed to facilitate the spin-orbit coupling, promote the intersystem crossing (ISC) [13–15], or restrain nonradiative transition [16], which include heavy atom effect [17], hydrogen bonding [18, 19], crystallization [20], host-guest complexation [21], and ionic bonding [22, 23]. Thus, many different types of inorganic materials with variable luminescence output have been explored, such as transition element complexes, inorganic semiconductor nanocrystals [24], carbon dots [25], and rare earth metal-organic frameworks [26]. While metal-containing compounds are efficient RTP luminophores, heavy metal complexes are often costly and highly biological toxic, with low processability as well as poor flexibility and good biocompatibility [26–30]. Compared to inorganic materials, some organic phosphorescence materials have the advantages of a wide variety, high compatibility, appreciable stability, and good processability [5, 31, 32]. Therefore, organic RTP materials possessing ultralong emission from their structural versatility are attractive alternatives.

Conventional single component RTP emission has normally an emission lifetime over 100 ms and quantum yield (*Φ*p) below 5% under ambient conditions [33, 34]. Some recently discovered long-lived RTP systems show *Φ*p of up to 31.2% (2,4,6-trimethoxy-1,3,5-triazine powder) [35] and a lifetime of more than 1,360 ms (isophthalic acid structure) [21, 23]. Studies have also been conducted to realize persistent RTP through single component organic materials and doped systems, which include a persistent luminescence material based on the organic photo-induced charge separation system [36], amide derivatives with high quantum yield and long lifetime [33], visible-light-excited organic green phosphorescence emission system [37], and single-crystal emission system from blue to red based on the diphenylsulfone core functionalized with phenoxazine and phenothiazine units [38]. Meanwhile, we recently reported a series of pyrene derivatives doped in polyvinyl alcohol (PVA) matrix to show tunable room-temperature polymer phosphorescence (RTPP) from blue to red [39] and dynamic excitation-dependent polymeric long persistent luminescence systems using polyphosphazenes [40]. Nevertheless, RTPP systems with ultralong lifetime and high quantum yield are still rare.

Although electronic medium has become an indispensable part of our daily life, the paper is still a widely used information storage medium. Therefore, the security requirements of paper information are demanding higher and higher. One of the most popular ways to achieve paper-based secure data recording is to use security ink, where printed information or patterns are visible only under ultraviolet light [41, 42]. While a series of photoluminescent material inks such as stimulus-responsive discolored systems, photochromic polymers, metal-free carbon dots, and ion quenched fluorescence switches have been reported [43, 44], fluorescent inks are mainly limited to single security with short fluorescence lifetime, which is unable to fulfill higher level security requirements.

**Figure 1:**
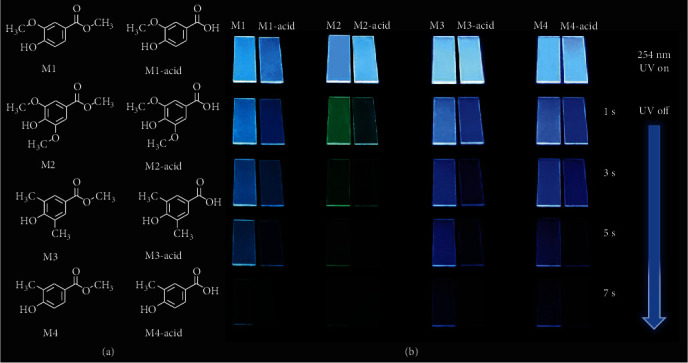
Chemical structures and long-lived phosphorescence photographs. (a) Chemical structures of M1, M1-acid, M2, M2-acid, M3, M3-acid, M4, and M4-acid. (b) Long-lived phosphorescence photographs of M-doped PVA films under 254 nm excitation.

Based on the molecular design viewpoint, hydrogen bonding interaction is very important for achieving RTPP by minimizing the nonradiative decay processes [45, 46]. Vanilla derivatives can form inter/intramolecular hydrogen bonding in the PVA matrix to suppress nonradiative transition or reduce molecular motions and triplet deactivation process by forming a relatively rigid amorphous environment (Figure 1). Herein, we tuned methoxy (-OCH_3_) and carboxylic acid (-COOH) functional groups on vanilla derivatives, where vanilla molecules surrounded by mutual hydrogen bonding interactions in the PVA matrix could lead to suppressed nonradiative transition. To validate this strategy, eight simple vanilla derivatives (M1 to M4 and M1-acid to M4-acid) were doped into the PVA matrix. Obviously, all films (except M2) emit sky blue phosphorescence emission after the removal of 254 nm UV lamp. These films exhibit strong RTPP performance with long lifetime phosphorescence emission, reaching 7 s emission under naked eye observation (Supplementary Figure S1 and Table S1). M4 with the PVA matrix shows the longest phosphorescence lifetime up to 369.8 ms, which is longer than most of the reported RTPP materials. It is noteworthy that the phosphorescent lifetime of M2 with the PVA matrix is 3.9 times higher than its acid form M2-acid in the PVA matrix. These results demonstrate a new method to discover more RTPP materials. Because of the selective emission characteristics of the paper matrix, the visualization for on/off switching of phosphorescence signals could be realized, showing promising potential for information encryption and fingerprint identification.

## 2. Results and Discussion

**Figure 2:**
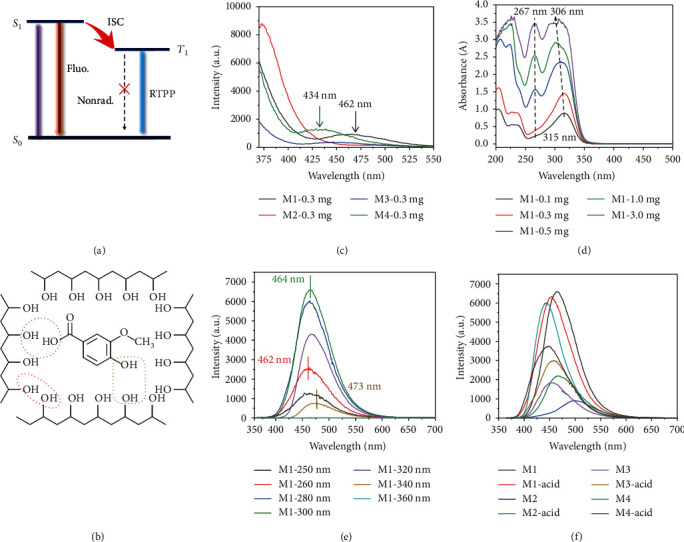
Schematic illustration and spectral studies. (a, b) Schematic illustration of the RTPP process and hydrogen bonding in vanilla-doped PVA films. Fluo.: fluorescence; Nonrad.: nonradiative transition. (c) Fluorescence spectra (*λ*_ex_ = 254 nm) of M1-M4-doped PVA at 0.3 mg mL^−1^ doping concentration. (d) UV-Vis absorption spectra of M1-doped PVA films at 0.1 mg, 0.3 mg, 0.5 mg, 1.0 mg, and 3.0 mg mL^−1^. (e) Phosphorescence spectra of M1-0.3 mg PVA film with 250 nm-360 nm excitation wavelength. (f) Phosphorescence spectra (*λ*_ex_ = 254 nm) of vanilla-doped PVA films at 0.3 mg mL^−1^ doping concentration.

To study the photophysical properties, a series of films were fabricated by a drop-coasting method based on hydrolyzed PVA aqueous solution (30 mg mL^−1^) containing different concentrations of vanilla compounds (0.1, 0.3, 0.5, 1.0, and 3.0 mg mL^−1^ denote as M-0.1 mg, M-0.3 mg, M-0.5 mg, M-1.0 mg, and M-3.0 mg, respectively). As PVA has a lot of hydroxy groups, these vanilla compounds with hydroxy groups could easily form hydrogen bonding with PVA. The formed hydrogen bonding interactions provide a relatively rigid environment to suppress nonradiative transition, beneficial to the generation of long-lived phosphorescence emission [18, 19]. Thus, hydrogen bonding is the main factor to suppress the total nonradiative decay from triplet excited state based on the intramolecular and intermolecular processes in vanilla-doped PVA films (Figures 2(a) and 2(b) and Supplementary Figure S2).

Among them, M1-M4-doped PVA films have a longer phosphorescence lifetime than that of M-acid-doped ones because of different substitutions. There are two major reasons to consider. (i) Because having more methyl groups would occupy more space and result in the suppression of nonradiative decay [47], M1 shows a longer phosphorescence lifetime than that of M1-acid in the PVA film. The intermolecular motion of the -COOH group is easier than that of -COOCH_3_, which may increase the *K*_nr_^Phos^ rate from the triplet excited state in the M-acid series. For example, while the chemical structures of M1 and M1-acid are very similar, the *K*_nr_^Phos^ of M1-acid is 3.5 times higher than that of M1 in the PVA film (Supplementary Figure S3). (ii) Energy dissipation in the form of thermal relaxation of the -OCH_3_ group is larger than that of the -CH_3_ group, which influences the phosphorescence lifetime. For example, M1-doped film (363.8 ms) has a longer phosphorescence lifetime than M2 (282.2 ms), M1-acid-doped film (101.3 ms) has a longer lifetime than M2-acid (72.8 ms), M4-doped film (369.8 ms) has a longer lifetime than M1 (363.8 ms), and M4-acid doped film (105.5 ms) has longer lifetime than M1-acid (101.3 ms).

Powder X-ray diffraction (XRD) studies show supporting evidence (Supplementary Figure S4). Pure PVA film exhibits two diffraction peaks at *θ*_1_ = 19.52° and *θ*_2_ = 22.44°. To our surprise, the diffraction peaks of M1 (*θ*_1_ = 19.32° and *θ*_2_ = 22.40°) and M1-acid (*θ*_1_ = 19.40° and *θ*_2_ = 22.50°) doped with PVA are different from that of the pure PVA film. A similar situation happens to other doped PVA films. These results indicate that different microstructures of these vanilla-doped PVA films have a different degree of hydrogen bonding formation, leading to the shifts of the two characteristic diffraction peaks in all doped films. Differential scanning calorimetry results exhibit that M1-0.3 mg has the highest glass transition temperature than other M1-doped PVA samples. Thermogravimetric analysis also exhibits that M1-0.3 mg has the highest temperature of 95% weight loss (Supplementary Figure S5), indicating an important role of hydrogen bonding interaction in the doped PVA. The effect of the hydroxyl group was also confirmed in different rigid matrices. It was proven that the amount of hydrogen bonding interactions in doped systems is closely related to the suppression of nonradiative processes (Supplementary Figure S6).

**Figure 3:**
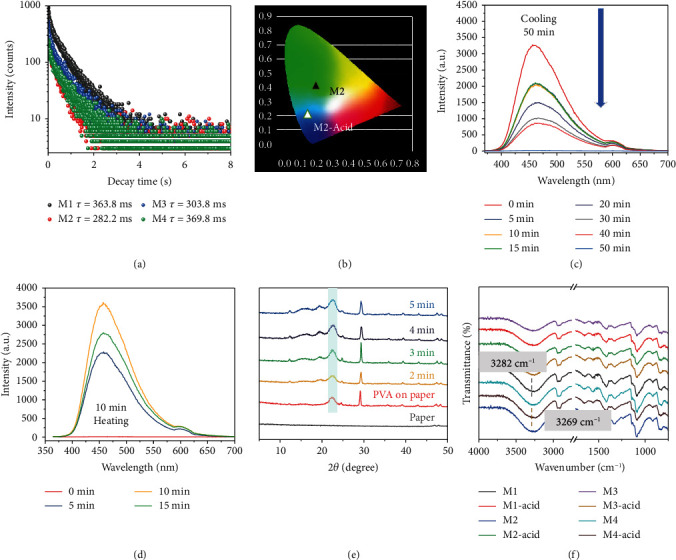
Phosphorescence spectra, powder XRD patterns, and FT-IR spectra. (a) Phosphorescence decay profiles of M1-M4-doped PVA. (b) CIE diagram for the emission spectra of M2 and M2-acid-doped PVA. (c,d) Phosphorescence emission spectra of the M1-doped PVA film with cooling and heating processes (*λ*_ex_ = 254 nm). (e) Powder XRD patterns of the M1-doped PVA film at heating conditions. (f) FT-IR spectra of these vanilla-doped PVA films.

The maximum emission peak of these vanilla-doped PVA locates around 460 nm (M4-doped PVA is at 435 nm). Under the excitation at 254 nm (Figure 2(c) and Supplementary Figure S7), M1-doped PVA shows an obvious fluorescence emission peak even at low concentrations (0.1 and 0.3 mg). To confirm the most suitable excited wavelength, UV-vis absorption spectra were obtained, indicating that M1-doped PVA has two different absorption peaks (Figure 2(d)). M1-0.1 mg and M1-0.3 mg only show the UV absorption at 315 nm, while M1-0.5 mg, M1-1.0 mg, and M1-3.0 mg systems present two new absorption peaks at 267 nm and 306 nm. At the same time, transmittance spectra indicate that all films possess good transparent properties (Supplementary Figure S8). Phosphorescence emission spectra of the M1-PVA film exhibit slight excitation-dependent emission under 250-360 nm excitation, and the maximum emission peak shifts from 462 nm to 473 nm upon changing the excitation from 250 nm to 340 nm (Figure 2(e) and Supplementary Figure S9a). Other doped PVA films were also investigated (Figure 2(f)). It should be noted that the maximum phosphorescence emission of M1-doped PVA film (465 nm) red shifts as compared with that of M1-acid-doped PVA film (453 nm). The reason might be ascribed to different electron delocalization of substituents (-COOCH_3_ and -COOH) on the phosphor molecules (Supplementary Figure S9b,c). M2-doped PVA film shows the copper green emission at 501 nm when compared with the M2-acid-doped PVA film, as indicated in the Commission Internationale de'L'eclairage (CIE) map (Figure 3(b)). The different RTPP emission ranges may be caused by different emission states (aggregated or separated) of phosphors in the PVA matrix.

Long-lived phosphorescence decay profiles show monoexponential fitting, indicating that only one triplet emission center exists for these films (Figure 3(a) and Supplementary Figure S10 and S11). Luminescent lifetime and intensity decrease with the increase of temperature from 77 K to 300 K, exhibiting typical RTP emission characteristics (Supplementary Figure S12-S14 and Table S2). For further understanding, the mechanism of the long-lived phosphorescence, the phosphorescence emission lifetime, and quantum yields of these films were recorded under ambient conditions. M4-doped PVA shows the longest phosphorescence lifetime (*τ* = 369.8 ms) with *Φ*_p_ of 11.19%, while M2-acid-doped PVA exhibits the shortest phosphorescence lifetime (*τ* = 72.8 ms) with *Φ*_p_ of 12.93%. It should be noted that, although M2-doped PVA has lower *Φ*_p_ than that of M2-acid-doped PVA, the former exhibits 4 folds of lifetime higher than that of the latter. Similarly, M3-doped PVA (*τ* = 303.8 ms) shows a longer phosphorescence lifetime than that of M3-acid-doped PVA (*τ* = 223.4 ms), and the phosphorescence lifetime of M4-doped PVA is longer than that of M4-acid-doped PVA. As compared with M4-doped PVA, M1 with one more -OCH_3_ group in the PVA matrix presents a longer lifetime, but lower *Φ*_p_. The reason may be that the -OCH_3_ group rotates more easily than -CH_3_, thus causing higher intermolecular motion. It is very interesting that these pure organic compounds in the PVA matrix show such a long phosphorescence lifetime with high quantum yields, attributed to the strong hydrogen bonding interaction for suppressing nonradiative transition in the doped systems. Taking M1 as an example (Supplementary Figure S2, Figure S3, and Table S1), the *K*_nr_^phos^ value of M1 at the crystal state and doped film state is 2258 s^−1^ and 2.56 s^−1^, respectively. For these doped PVA films, the RTPP is dominated by the doping concentration of phosphors. An optimum concentration of vanilla-doped PVA was determined to be 0.3 mg mL^−1^ of vanilla compounds in the PVA aqueous solution (30 mg mL^−1^). At lower doping concentrations, vanilla compounds could not form enough hydrogen bonding with PVA to suppress the nonradiative transition. When the doping concentration is over 0.5 mg mL^−1^, excessive -COOH and/or -OH groups of phosphors cannot fully form hydrogen bonding with the PVA chain, thus increasing the vibration of the PVA system. Therefore, it is generally appreciated that photophysical properties are closely related to the molecular structure of these phosphors.

Interestingly, the phosphorescence property of these vanilla-doped PVA films shows obvious temperature dependence. The major attraction is the significant change of phosphorescence spectra upon naturally cooling from 65°C to room temperature under ambient conditions, where the maximum phosphorescence emission intensity decreases from 3,250 to almost 0 a.u. after 50 min (Figure 3(c)). By heating the samples under the same conditions, obvious phosphorescence intensity could be recovered (Figure 3(d)). In addition, M1-0.3 mg-doped film exhibits almost identical phosphorescence intensity in different environments (i.e., air, argon, and oxygen), revealing the inertness of the triplet excited states to oxygen (Supplementary Figure S15a). However, the phosphorescence emission spectra are sensitive to water molecule (Supplementary Figure S15b). On account of hydrogen bonding interaction in these RTPP systems, the temperature dependence should be caused by moisture in the air, i.e., water could permeate into films to break hydrogen bonding between vanilla molecules and PVA matrix [5]. During the cooling process, hydrogen bonding interactions would be destroyed by the interference of the water molecules, resulting in increased nonradiative transition pathways in doped systems. On the contrary, in the heating process, the evaporation of water molecules from the doping systems leads to the reformation of hydrogen bonding interactions between phosphors and PVA.

To gain a deep insight into the unique phosphorescence, the UV stability experiments of all luminescent films were conducted (Supplementary Figure S16). The maximum emission intensity shows a very little change when the irradiation time increases from 0 to 20 hours, revealing that the UV stability of these long-lived room-temperature polymer phosphorescence films is high. In addition, weak phosphorescence emission of M1-0.3 mg-doped PMMA also proved that a relatively rigid amorphous matrix provided by PVA is necessary for vanilla phosphors (Supplementary Figure S17). Powder XRD studies provide further evidence for the temperature dependence of the films (Supplementary Figure S18). A new diffraction peak at about *θ* = 22° for M1-0.3 mg PVA film appears under gentle heating (Figure 3(e)), because of the reformation of hydrogen bonding after the water removal. On the contrary, no diffraction peak appears at the same position (Supplementary Figure S18b) during the cooling process. These studies also indicate that more hydrogen bonding interactions are formed with the increase of M1 phosphor concentration in the doped PVA film (Supplementary Figure S18c). Hydrogen bonding interaction between the matrix and phosphors was proven circumstantially by Fourier-transform infrared (FT-IR) spectroscopy. The vibration of the -OH group in pure PVA matrix locates at 3,252 cm^−1^, attributed to intermolecular and intramolecular hydrogen bonding interactions (Figure 3(f) and Supplementary Figure S19). The -OH vibration of vanilla-doped PVA films shows upward shifts of 6-24 cm^−1^ (24 cm^−1^ for the M1-doped PVA film and 6 cm^−1^ for M2-doped PVA). These results clearly validate that the vanilla phosphors have a strong ability to form hydrogen bonding interaction with PVA.

**Figure 4:**
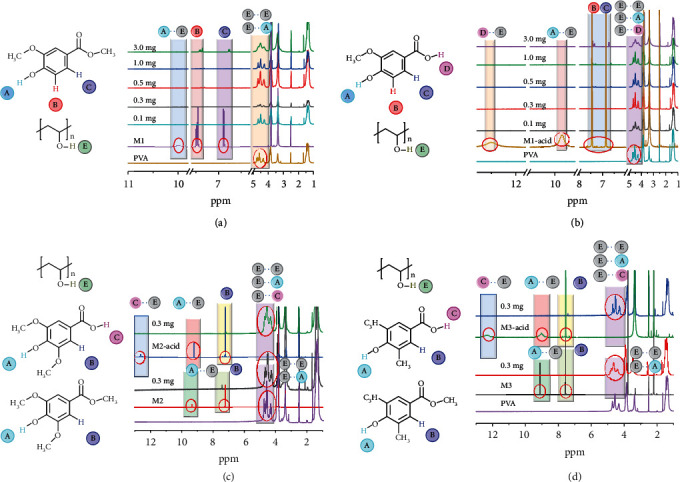
^1^H NMR spectral studies. ^1^H NMR spectra of (a) M1, (b) M1-acid, (c) M2 and M2-acid, and (d) M3 and M3-acid-doped PVA films with different doping concentrations. On the left: vanilla structures with indicated protons and PVA structure with E proton. On the right: ^1^H NMR spectra showing the changes of proton peaks.

To further probe into the nature of obvious RTPP properties from these films under ambient conditions, proton nuclear magnetic resonance (^1^H NMR) spectra were carried out (Figure 4). Taking M1-acid as an example, its protons are marked as A-D, and -OH of PVA is labelled as E in Figure 4(b). Proton D from -COOH located at 12.5 ppm completely disappears after doping M1-acid with PVA at low concentrations, attributed to D-E type hydrogen bonding. A similar situation occurs to proton A from -OH located at 9.86 ppm, with A-E type hydrogen bonding. Besides, three peaks from isotactic, heterotactic, and syndiotactic structures of the -OH group at 4.3 ppm to 4.6 ppm become a broad peak at 4.5 ppm after forming the M1-acid-3.0 mg PVA film. Upon increasing the doping concentration, protons B and C on the benzene ring gradually appear, along with a red shift. At high concentrations, those initially disappeared proton peaks recover. A possible explanation is that PVA cannot provide enough hydroxy groups to bind with vanilla molecules at high concentrations. ^1^H NMR spectra of other doped films show a similar trend (Supplementary Figure S20). These observations prove the formation of strong intermolecular hydrogen bonding between vanilla compounds and PVA matrix.

**Figure 5:**
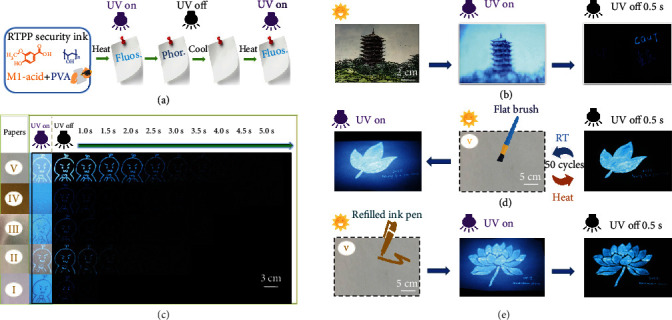
Painting, reading, and erasing processes of the RTPP ink. (a) Controllable RTPP emission process of M1-acid-0.3 mg PVA ink on a substrate. (b) Sensitive anticounterfeiting on a postcard after turning off 254 nm light. (c) Comparison of the ink emission observed from five different paper substrates. (d) Photos of ink-brushed leaf pattern on glazed printing paper, showing temperature-dependent changes between room temperature (RT) and 65°C for 50 times. Bright blue fluorescence emits when turning on the 254 nm UV lamp, and cobalt phosphorescence emission is observed when turning off the 254 nm UV lamp. (e) A hand-painted lotus flower pattern with M1-acid-0.3 mg PVA ink showing cobalt phosphorescence emission after heating treatment (65°C) and turning of the UV light.

Unique RTP property and sensitive RTPP character of M1-acid-0.3 mg PVA make it an attractive ink material in advanced anticounterfeiting and information storage since such a long-lived excited state is very difficult to be replicated. The rapid anticounterfeiting processes of phosphorescent ink pen made using M1-acid-0.3 mg PVA are illustrated in Figure 5(a), where the fluorescence and phosphorescence emission on paper could be carefully controlled. As shown from Figure 5(b) and Supplementary Video S1, the “CQUT” letters on the postcard were written just once, and invisible information could be easily read by the naked eye after turning off the UV lamp. Long-lived phosphorescence emission could be recognized more than 3 s after removing the UV lamp, indicating that the RTPP ink can be used to hand-write on the postcard substrate. To further explore more abundant applications, we carried out this on/off visualization with different substrates, including offset papers, parchment, white card papers, kraft papers, and glazed printing papers, marked as I-V, respectively. M1-acid-0.3 mg PVA was used as the RTPP pen ink to paint on these five types of paper substrates (Figure 5(c) and Supplementary Video S2). Surprisingly, the RTPP ink is highly selective to the substrates, showing different RTP intensities and phosphorescence emission time. The longest emission about 5 s is from paper V, and paper I exhibits the shortest RTPP emission (still over 1 s). The emission difference may be due to the different composition and cellulose contents of these papers. The ink on these papers could still be distinguishable regardless of weak blue fluorescence interference from the paper background, showing its high application potential for multiple information anticounterfeiting.

Powder XRD and FT-IR analyses prove different RTPP emissions on these paper substrates, attributed to different hydrogen bonding interactions as previously demonstrated. In powder XRD patterns, the peak of the blank paper V at *θ* = 28.47° gradually increases to 29.52° after painting with PVA solution, indicating that the hydrogen bonding formation takes effect on paper V. Meanwhile, FT-IR spectra also show the stabilizing process by weakening the vibration of -OH in paper V (Supplementary Figure S21). Offset paper and parchment exhibit a similar stabilization process through hydrogen bonding interaction (Supplementary Figure S22). Atomic force microscopy (AFM) studies also demonstrate no variation of roughness on the surface of encrypted papers (Supplementary Figure S23). The tensile test shows that doped vanilla compounds have a certain effect on the toughness of the films when increasing the doping concentrations (Supplementary Figure S23). The results indicate that the pure PVA film has the highest tensile strength of 25.61 MPa. The tensile strength of the M1-0.3 mg PVA film under the best luminescence intensity is 19.66 MPa, still having a good ductility.

Herein, we show how to use a flat brush to brush the security ink on the papers and refill the ink into a pen. We make exquisite patterns with highly efficient identification and double emission (Figures 5(d) and 5(e) and Supplementary Video S3 and Video S4). The preparation process is very simple and convenient. The results in Figure 5(d) indicate that this kind of long-lived phosphorescence emission ink could be used in a large area, achieving very strong fluorescence and phosphorescence dual emission on the papers. After heating and cooling treatments based on the procedure shown in Figure 5(a) for 50 cycles, excellent luminescence performance could still be maintained, demonstrating the high robustness of the ink. Refilling the RTPP ink into an empty signing pen is highly feasible and reproducible (Figure 5(e)). We draw a lotus flower picture only once on the white card paper with the RTPP ink pen. What excites us is that the pattern with double emission characteristics drawn by hand is even more exquisite than that drawn by the painting brush, which would lay a foundation for the generalization of the RTPP ink pen in the future.

## 3. Conclusion

In conclusion, we have fabricated a series of efficient RTPP systems by doping simple vanilla derivatives into PVA through hydrogen bonding interactions, achieving the maximum phosphorescence lifetime of 369.8 ms and the maximum phosphorescence quantum yield of 14.36%. Because of unique photophysical properties with long-lived phosphorescence emission, these RTPP systems could be employed as security inks. The security inks could be widely utilized on different substrates, with the longest emission over 5 s on the glazed printing paper. The prepared security inks not only maintain excellent luminescent properties under ambient conditions but also exhibit highly identifiable characters after repeated heating/cooling cycles for at least 50 times. Owing to these remarkable features of the RTPP inks, an advanced anticounterfeiting data encoding/reading strategy based on handwriting technology and complex pattern steganography has been proposed for future practical uses.

## 4. Experimental Methods

### 4.1. Preparation of Doped Matrix

PVA solid (6 g) with hydrolysis degree of 100, 89, and 80 was, respectively, dissolved in deionized water (200 mL) at 95°C for 1 h, which was then filtered to obtain PVA aqueous solution (30 mg mL^−1^) for further use. Poly(methyl methacrylate) solid (3 g) was dissolved in tetrahydrofuran (100 mL) at 65°C for 1 h and then filtered for further use.

### 4.2. Preparation of Vanilla-Doped Films and Phosphorescent Inks

Firstly, M1, M1-acid, M2, M2-acid, M3, M3-acid, M4, and M4-acid (0.3 mg mL^−1^ for each molecule) were, respectively, dispersed in eight vials of PVA solutions with the concentration of 30 mg mL^−1^, and eight homogeneous solutions were obtained after ultrasonication for 3 h. Secondly, a series of films were fabricated by a drop-coasting method using each aqueous solution (30 mg mL^−1^) containing different concentrations of vanilla compounds. Meanwhile, these eight solutions were, respectively, injected into eight pen cartridges to yield eight security inks. Thirdly, a hand-painted lotus and ink-brushed leaf were prepared by hand painting. Fourthly, after drying in air, the patterns were dried under 65°C in an oven for 1 h.

## Data Availability

All data needed to evaluate the conclusions in the paper are present in the paper and the supplementary materials. Additional data related to this paper may be requested from the authors.

## Supplementary Materials

Supplementary Materials Materials, measurements, and photoluminescence quantum yield. Figure S1: phosphorescence emission lifetime of vanilla- (0.3 mg-) doped PVA (phosphorescence time: ms). Figure S2: (a) phosphorescence emission decay profiles of these vanilla derivatives in the crystal state at room temperature. Quantum yield of M1 crystal: 0.64%. (b) Phosphorescence emission of M1 in its crystal state (M1-Crystal) and M1- (0.3 mg-) doped film at 77 K. Figure S3: nonradiative decay rate of phosphorescence (*K*_nr_^phos^) for vanilla derivatives (0.3 mg) doped with PVA matrix. Figure S4: powder XRD patterns of vanilla-doped PVA films with 0.3 mg doping concentration. Figure S5: (a) DSC curves of M1-doped PVA films with different doping concentrations. (b) TGA curves of M1-doped PVA films with different doping concentrations. (c) DSC curves of M1-M4 doped PVA films with 0.3 mg doping. (d) DSC curves of M1-acid-M4-acid-doped PVA films with 0.3 mg doping. Figure S6: phosphorescence spectra of M1- (0.3 mg-) doped PVA-100, PVA-87, PVA-80, and PMMA. Figure S7: (a) fluorescence spectra of M1-acid to M4-acid-doped PVA at 0.3 mg doping concentration. (b) fluorescence spectra of M1-doped PVA with different doping concentrations. Figure S8: (a) transmission spectra of the M1-doped PVA film with different doping concentrations. (b) Transmission spectra of the pure PVA film and vanilla-doped PVA films with 0.3 mg doping concentration. (c) UV absorption spectra of the pure PVA film and vanilla-doped PVA films with 0.3 mg doping concentration. Figure S9: (a) phosphorescence spectra of M1-doped PVA with different doping concentrations. (b, c) Steady state fluorescence (red dashed line) and long-lived phosphorescence (blue solid line) spectra of M1 and M1-acid-doped PVA. Figure S10: fluorescence lifetime of M1- (0.3 mg-) doped in PVA (M1-0.3 mg-PVA) and in the crystal state (M1-crystal) at ambient conditions. M1-0.3 mg-PVA: @ 364 nm; M1-crystal: @ 384 nm. Figure S11: (a) phosphorescence decay profiles of M1-doped PVA films with different doping concentrations. (b) Phosphorescence decay profiles of M1-doped PVA films with different excitation wavelengths. (c) Phosphorescence decay profiles of M1-M4 at 0.3 mg concentration. Figure S12: temperature-dependent (a) luminescence decay curves and (b) phosphorescence emission spectra for M1- (0.3 mg-) doped film. Figure S13: (a)–(h) steady state fluorescence (black line) and long-lived phosphorescence (red line) spectra of vanilla-doped PVA at 0.3 mg doping concentration at 77 K. Figure S14: (a)–(h) Steady state fluorescence (black line) and long-lived phosphorescence (red line) spectra of vanilla-doped PVA at 0.3 mg doping concentration at room temperature. Figure S15: phosphorescence emission spectra of the M1- (0.3 mg-) doped PVA film (a) in different environments at room temperature (air, argon, and oxygen) and (b) under different fumigating times by commercial humidifier. Figure S16: phosphorescence emission spectra of the M1-acid- (0.3 mg-) doped PVA film under different irradiation times by UV 254 nm at room temperature. Figure S17: phosphorescence emission of M1 (0.3 mg) doped in PMMA and PVA matrix under dry argon atmosphere. Figure S18: (a) powder XRD patterns of vanilla derivatives in solid state. (b) Powder XRD patterns showing the cooling process of the M1-doped PVA film at 0.3 mg doping concentration. (c) Powder XRD patterns of M1-doped PVA films with different doping concentrations. Figure S19: (a) FT-IR spectra of vanilla-doped PVA. (b) FT-IR spectra of M1-doped PVA with different doping concentrations. Figure S20: ^1^H NMR spectra of M4 and M4-acid-doped PVA in DMSO-d_6_. Left: M4 structure with four H protons (a)–(d), M4-acid structure with three H protons (a)–(c), and PVA structure with E proton. Right: ^1^H NMR spectra showing the changes of different H protons. Figure S21: (a) FT-IR spectra of blank paper I, paper with 30 mg mL^−1^ PVA, and paper with M1-acid-0.3 mg PVA. (b) FT-IR spectra of blank paper II, paper with 30 mg mL^−1^ PVA, and paper with M1-acid-0.3 mg PVA. (c) FT-IR spectra of blank paper III, paper with 30 mg mL^−1^ PVA, and paper with M1-acid-0.3 mg PVA. (d) FT-IR spectra of blank paper IV, paper with 30 mg mL^−1^ PVA, and paper with M1-acid-0.3 mg PVA. Figure S22: (a) phase scanning XRD patterns of blank paper I, paper with 30 mg mL^−1^ PVA, and paper with M1-acid-0.3 mg PVA. (b) Phase scanning XRD patterns of blank paper II, paper with 30 mg mL^−1^ PVA, and paper with M1-acid-0.3 mg PVA. (c) Phase scanning XRD patterns of blank paper III, paper with 30 mg mL^−1^ PVA, and paper with M1-acid-0.3 mg PVA. (d) Phase scanning XRD patterns of blank paper IV, paper with 30 mg mL^−1^ PVA, and paper with M1-acid-0.3 mg PVA. (e) Phase scanning XRD patterns of blank paper V, paper with 30 mg mL^−1^ PVA, and paper with M1-acid-0.3 mg PVA. (f) FT-IR spectra of blank paper V, paper with 30 mg mL^−1^ PVA, and paper with M1-acid-0.3 mg PVA. Figure S23: AFM surface topography of vanilla-doped PVA inks with 0.3 mg doping concentration applied on paper V. Scale: 5 × 5 *μ*m. Figure S24: tensile tests of M1-doped PVA films with different doping concentrations. (a) Film stretching process. (b) Tensile curves of M1-doped PVA films. Table S1: fluorescence quantum yields (*Φ*_f_), phosphorescence lifetime (*τ*_p_), and quantum yields (*Φ*_p_) of M1, M1-acid, M2, M2-acid, M3, M3-acid, M4, and M4-acid-doped PVA films. Table S2: singlet-triplet energy gap (*Δ*E_ST_) of vanilla derivatives doped with the PVA film at 0.3 mg doping concentration. Video S1 (.avi format): emission of M1-acid-0.3 mg-doped PVA ink applied on postcard when the UV light is on and off. Video S2 (.avi format): emission of M1-acid-0.3 mg-doped PVA ink applied on paper V when the UV light is on and off. Video S3 (.avi format): emission of M1-0.3 mg-doped PVA ink brushed on paper V when the UV light is on and off. Video S4 (.avi format): emission of M1-0.3 mg-doped PVA ink hand-drawn on paper V when the UV light is on and off. See References [48–50].
