# Supplementary material for: Simple Vanilla Derivatives for Long-Lived Room-Temperature Polymer Phosphorescence as Invisible Security Inks
Source: Research (Wash D C). 2021 Feb 16;2021:8096263. doi: 10.34133/2021/8096263 (PMC7906025; doi:10.34133/2021/8096263)
Supplement: Supplementary Materials — Materials, measurements, and photoluminescence quantum yield. Figure S1: phosphorescence emission lifetime of vanilla- (0.3 mg-) doped PVA (phosphorescence time: ms). Figure S2: (a) phosphorescence emission decay profiles of these vanilla derivatives in the crystal state at room temperature. Quantum yield of M1 crystal: 0.64%. (b) Phosphorescence emission of M1 in its crystal state (M1-Crystal) and M1- (0.3 mg-) doped film at 77 K. Figure S3: nonradiative decay rate of phosphorescence (Knrphos) for vanilla derivatives (0.3 mg) doped with PVA matrix. Figure S4: powder XRD patterns of vanilla-doped PVA films with 0.3 mg doping concentration. Figure S5: (a) DSC curves of M1-doped PVA films with different doping concentrations. (b) TGA curves of M1-doped PVA films with different doping concentrations. (c) DSC curves of M1-M4 doped PVA films with 0.3 mg doping. (d) DSC curves of M1-acid-M4-acid-doped PVA films with 0.3 mg doping. Figure S6: phosphorescence spectra of M1- (0.3 mg-) doped PVA-100, PVA-87, PVA-80, and PMMA. Figure S7: (a) fluorescence spectra of M1-acid to M4-acid-doped PVA at 0.3 mg doping concentration. (b) fluorescence spectra of M1-doped PVA with different doping concentrations. Figure S8: (a) transmission spectra of the M1-doped PVA film with different doping concentrations. (b) Transmission spectra of the pure PVA film and vanilla-doped PVA films with 0.3 mg doping concentration. (c) UV absorption spectra of the pure PVA film and vanilla-doped PVA films with 0.3 mg doping concentration. Figure S9: (a) phosphorescence spectra of M1-doped PVA with different doping concentrations. (b, c) Steady state fluorescence (red dashed line) and long-lived phosphorescence (blue solid line) spectra of M1 and M1-acid-doped PVA. Figure S10: fluorescence lifetime of M1- (0.3 mg-) doped in PVA (M1-0.3 mg-PVA) and in the crystal state (M1-crystal) at ambient conditions. M1-0.3 mg-PVA: @ 364 nm; M1-crystal: @ 384 nm. Figure S11: (a) phosphorescence decay profiles of M1-do [file 8096263.f1.zip › 8096263.f1/Revised Supplementary Materials.docx]

**Supplementary Materials**

# Simple Vanilla Derivatives for Long-Lived Room Temperature Polymer Phosphorescence as Invisible Security Inks

Yongfeng Zhang,^1,†^ Zhonghao Wang,^1,†^ Yan Su,^1^ Yan Zheng,^1^ Wenji Tang,^1^ Chaolong Yang,^1,2^* Hailong Tang,^1^ Lunjun Qu,^1^ Youbing Li,^1^ Yanli Zhao^2,^*

1School of Materials Science and Engineering, Chongqing University of Technology, Chongqing, 400054

2Division of Chemistry and Biological Chemistry, School of Physical and Mathematical Sciences, Nanyang Technological University, 21 Nanyang Link, Singapore 637371

^†^These authors contributed equally to this work.

*Correspondence should be addressed to Chaolong Yang; yclzjun@163.com and Yanli Zhao; zhaoyanli@ntu.edu.sg

**Materials:**

Unless other noted, all materials were purchased from commercial sources without further purifications. Polyvinyl alcohol (PVA, molecular weight (Mw) = 85,000 to 124,000 g mol^-1^, 100% hydrolyzed) was purchased from Sigma-Aldrich.

**Measurements:**

Fourier-transform infrared (FT-IR) spectra were carried out using a Nicolet Is-10 (Nicolet) Fourier Transform Infrared Spectrometer. Differential scanning calorimetry (DSC) was conducted on TA Q20 at a heating rate of 10 °C/min under nitrogen atmosphere. Thermogravimetric analysis (TGA, Q500) was carried out in nitrogen atmosphere from 30 to 700 °C with a ramping rate of 15 °C min^−1^. NMR spectra were taken on a DRX-400 MHz (Bruker) superconducting-magnet NMR spectrometer with tetramethylsilane as an internal standard. UV-vis absorption spectra were determined on a Shimadzu UV-3600 UV-Vis-NIR spectrophotometer. Fluorescence spectra, phosphorescent spectra, and lifetime were carried out on a on Edinburgh FLS1000 fluorescence spectrophotometer, and the samples were measured in the film state. Phosphorescence mode: total decay time 8 s, delay time 10 ms. Photoluminescence quantum efficiency was obtained on Edinburgh FLS1000 fluorescence spectrophotometer equipped with an integrating sphere. The photos and supporting videos were recorded by a Canon EOS 80D camera. AFM surface topography images were performed on NX10 (Parks). Powder X-ray diffraction (XRD) measurements were recorded on a Rigaku D/Max-2500 using Cu K_α_ radiation with 2θ range of 5 - 50°, 40 KeV, and 30 mA having a scanning rate of 0.01° s^−1^ (2θ) at room temperature. Tensile test measurements were recorded by CMY010L (Metis). The plastic film of 76 mm × 25 mm × 0.05 mm was prepared on a glass sheet by tape casting method. The tensile rate was 10 mm/min. The film was tested at room temperature.

**Photoluminescence quantum yield:**

By referring to the Edinburgh manual, we measured relative photoluminescence quantum yields (Φ_p_) by “direct excitation” method. The measurements of the sample emission were conducted in a position where it is directly excited by excitation beam in this method.

Excitation wavelength of M1, M2, M3, M4, M1-acid, M2-acid, M3-acid, and M4-acid doped PVA films is 315 nm, 304 nm, 348 nm, 309 nm, 311 nm, 300 nm, 315 nm, and 298 nm, respectively. The Φ_p_ test was conducted using a microsecond lamp as the excitation source (delayed time: 5 ms), and the fluorescence section can be filtered out by using the gating delay system, so that relatively more accurate Φ_p_ was obtained. The Φ_p_ measurements on FLS1000 were auto-calculated mainly based on different phosphorescence emission spectra of reference and samples. Φ_p_ was calculated according to the following formula provided by FLS1000 software.

$$\phi_{p}=\frac{E_{B}-E_{A}}{S_{A}-S_{B}}$$

where E_A_ is the luminescence integral region of reference, E_B_ is the phosphorescence integral region of sample, and S_A_ and S_B_ are the excitation scatter integral regions of reference and sample, respectively.

The photophysical parameters in this work were calculated using the following equations [48-50]:

$K_{r}^{Fluo}=\frac{\emptyset_{Fluo}}{\tau_{Fluo}}$ (1)

$K_{nr}^{Fluo}=\frac{{(1-\emptyset}_{Fluo}{-\emptyset}_{Phos})}{\tau_{Fluo}}$ (2)

$K_{ISC}=\frac{\emptyset_{Phos}}{\tau_{Fluo}}$ (3)

$K_{r}^{Phos}=\frac{\emptyset_{Phos}}{\tau_{Phos}}$ (4)

$K_{nr}^{Phos}=\frac{{(1-\emptyset}_{Phos})}{\tau_{Phos}}$ (5)

where K_r_^Fluo^ is fluorescence radiative rate constant, K_nr_^Fluo^ is fluorescence non-radiative rate constant, K_ISC_ is ISC rate constant, K_r_^Phos^ is phosphorescence radiative rate constant, and K_nr_^Phos^ is phosphorescence non-radiative rate constant.

**Supplementary Figures and Tables**

**Figure S1.** Phosphorescence emission lifetime of vanilla (0.3 mg) doped PVA (phosphorescence time: ms).

_

_

**Figure S2.** (a) Phosphorescence emission decay profiles of these vanilla derivatives in the crystal state at room temperature. Quantum yield of M1 crystal: 0.64 %. (b) Phosphorescence emission of M1 in its crystal state (M1-Crystal) and M1 (0.3 mg) doped film at 77 K.





**Figure S3**. Nonradiative decay rate of phosphorescence (*K*_nr_^phos^) for vanilla derivatives (0.3 mg) doped with PVA matrix.





**Figure S4.** Powder XRD patterns of vanilla doped PVA films with 0.3 mg doping concentration.





**Figure S5.** (a) DSC curves of M1 doped PVA films with different doping concentrations. (b) TGA curves of M1 doped PVA films with different doping concentration. (c) DSC curves of M1-M4 doped PVA films with 0.3 mg doping. (d) DSC curves of M1-acid - M4-acid doped PVA films with 0.3 mg doping.





**Figure S6.** Phosphorescence spectra of M1 (0.3 mg) doped PVA-100, PVA-87, PVA-80, and PMMA.





**Figure S7.** (a) Fluorescence spectra of M1-acid to M4-acid doped PVA at 0.3 mg doping concentration. (b) Fluorescence spectra of M1-doped PVA with different doping concentrations.





**Figure S8.** (a) Transmission spectra of M1-doped PVA film with different doping concentrations. (b) Transmission spectra of pure PVA film and vanilla-doped PVA films with 0.3 mg doping concentration. (c) UV absorption spectra of pure PVA film and vanilla-doped PVA films with 0.3 mg doping concentration.





**Figure S9.** (a) Phosphorescence spectra of M1-doped PVA with different doping concentrations. (b,c) Steady state ﬂuorescence (red dashed line) and long-lived phosphorescence (blue solid line) spectra of M1 and M1-acid doped PVA.


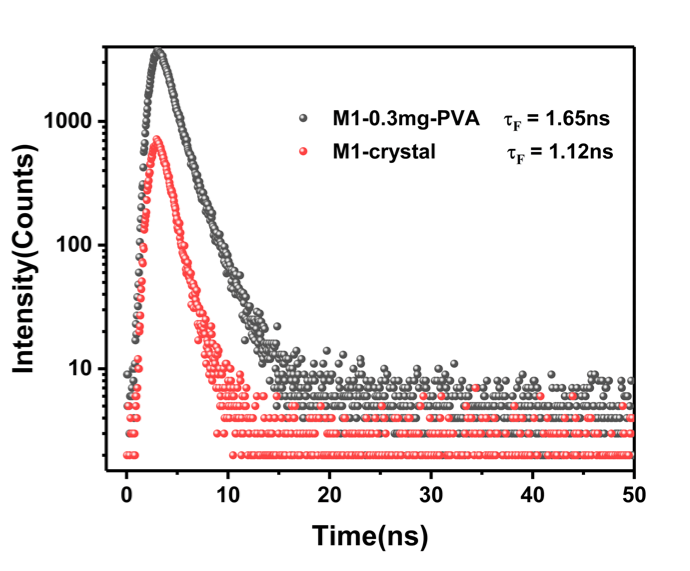


**Figure S10.** Fluorescence lifetime of M1 (0.3 mg) doped in PVA (M1-0.3mg-PVA) and in the crystal state (M1-crystal) at ambient conditions. M1-0.3mg-PVA: @ 364 nm; M1-crystal: @ 384 nm.





**Figure S11.** (a) Phosphorescence decay profiles of M1-doped PVA films with different doping concentrations. (b) Phosphorescence decay profiles of M1-doped PVA films with different excitation wavelengths. (c) Phosphorescence decay profiles of M1-M4 at 0.3 mg concentration.





**Figure S12**. Temperature-dependent (a) luminescence decay curves and (b) phosphorescence emission spectra for M1 (0.3 mg) doped film.





**Figure S13**. (a-h) Steady state fluorescence (black line) and long-lived phosphorescence (red line) spectra of vanilla doped PVA at 0.3 mg doping concentration at 77 K.





**Figure S14**. (a-h) Steady state fluorescence (black line) and long-lived phosphorescence (red line) spectra of vanilla doped PVA at 0.3 mg doping concentration at room temperature.

**Figure S15**. Phosphorescence emission spectra of M1 (0.3 mg) doped PVA film (a) in different environments at room temperature (air, argon, and oxygen), and (b) under different fumigating times by commercial humidifier.





**Figure S16.** Phosphorescence emission spectra of M1-acid (0.3 mg) doped PVA film under different irradiation times by UV 254 nm at room temperature.





**Figure S17.** Phosphorescence emission of M1 (0.3 mg) doped in PMMA and PVA matrix under dry argon atmosphere.





**Figure S18.** (a) Powder XRD patterns of vanilla derivatives in solid state. (b) Powder XRD patterns showing cooling process of M1 doped PVA film at 0.3 mg doping concentration. (c) Powder XRD patterns of M1 doped PVA films with different doping concentrations.





**Figure S19.** (a) FT-IR spectra of vanilla-doped PVA. (b) FT-IR spectra of M1-doped PVA with different doping concentrations.


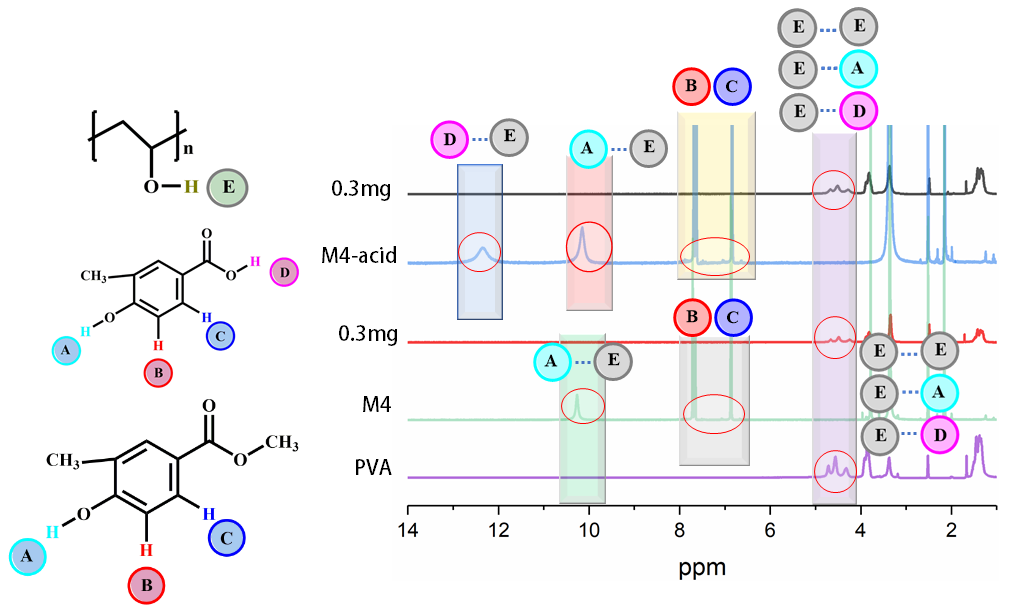


**Figure S20.** ^1^H NMR spectra of M4 and M4-acid doped PVA in DMSO-d_6_. Left: M4 structure with four H protons (A-D), M4-acid structure with three H protons (A-C), and PVA structure with E proton. Right: ^1^H NMR spectra showing the changes of different H protons.





**Figure S21.** (a) FT-IR spectra of blank paper I, paper with 30 mg mL^-1^ PVA, and paper with M1-acid-0.3 mg PVA. (b) FT-IR spectra of blank paper II, paper with 30 mg mL^-1^ PVA, and paper with M1-acid-0.3 mg PVA. (c) FT-IR spectra of blank paper III, paper with 30 mg mL^-1^ PVA, and paper with M1-acid-0.3 mg PVA. (d) FT-IR spectra of blank paper IV, paper with 30 mg mL^-1^ PVA, and paper with M1-acid-0.3 mg PVA.





**Figure S22.** (a) Phase scanning XRD patterns of blank paper I, paper with 30 mg mL^-1^ PVA, and paper with M1-acid-0.3 mg PVA. (b) Phase scanning XRD patterns of blank paper II, paper with 30 mg mL^-1^ PVA, and paper with M1-acid-0.3 mg PVA. (c) Phase scanning XRD patterns of blank paper III, paper with 30 mg mL^-1^ PVA, and paper with M1-acid-0.3 mg PVA. (d) Phase scanning XRD patterns of blank paper IV, paper with 30 mg mL^-1^ PVA, and paper with M1-acid-0.3 mg PVA. (e) Phase scanning XRD patterns of blank paper V, paper with 30 mg mL^-1^ PVA, and paper with M1-acid-0.3 mg PVA. (f) FT-IR spectra of blank paper V, paper with 30 mg mL^-1^ PVA, and paper with M1-acid-0.3 mg PVA.





**Figure S23.** AFM surface topography of vanilla doped PVA inks with 0.3 mg doping concentration applied on paper V. Scale: 5 × 5 μm.





**Figure S24.** Tensile tests of M1-doped PVA films with different doping concentrations. (a) Film stretching process. (b) Tensile curves of M1 doped PVA films.

**Table S1.** Fluorescence quantum yields (Φ_f_), Phosphorescence lifetime (τ_p_) and quantum yields (Φ_p_) of M1, M1-acid, M2, M2-acid, M3, M3-acid, M4, and M4-acid doped PVA films.

| Compounds | M1 | M1-acid | M2 | M2-acid | M3 | M3-acid | M4 | M4-acid |
| --- | --- | --- | --- | --- | --- | --- | --- | --- |
| Φ_f_ (%) | 34.72 | 49.48 | 34.42 | 29.23 | 38.05 | 38.33 | 33.29 | 57.07 |
| τ_f_ (ns) | 1.65 | 1.46 | 2.97 | 2.30 | 1.83 | 2.14 | 1.79 | 1.35 |
| τ_p_ (ms) | 363.8 | 101.3 | 282.2 | 72.8 | 303.8 | 233.4 | 369.8 | 105.5 |
| Φ_p_ (%) | 6.72 | 9.38 | 14.36 | 12.93 | 13.20 | 10.43 | 11.19 | 10.15 |

Note: the excitation wavelength of M1, M1-acid, M2, M2-acid, M3, M3-acid, M4, and M4-acid doped PVA films is 315 nm, 304 nm, 348 nm, 309 nm, 311 nm, 300 nm, 315 nm, and 298 nm, respectively. The emission wavelength for the phosphorescence lifetime is monitored at 465 nm, 453 nm, 501 nm, 458 nm, 430 nm, 410 nm, 435 nm, and 419 nm, respectively.

**Table S2**. Singlet-triplet energy gap (ΔE_ST_) of vanilla derivatives doped with PVA film at 0.3 mg doping concentration.

| Compounds | M1 | M1-aicd | M2 | M2-aicd | M3 | M3-aicd | M4 | M4-aicd |
| --- | --- | --- | --- | --- | --- | --- | --- | --- |
| ΔE_ST_ (eV) | 0.78 | 1.09 | 0.72 | 1.20 | 1.20 | 0.99 | 0.84 | 1.14 |
